# Dataset of metabolites extracted from African walnut (*Tetracarpidium conophorum*) using two different solvents

**DOI:** 10.1016/j.dib.2023.108930

**Published:** 2023-01-26

**Authors:** Beatrice Mofoluwaso Oladimeji, Oluwafemi Ayodeji Adebo

**Affiliations:** Food Innovation Research Group, Department of Biotechnology & Food Technology, Faculty of Science, University of Johannesburg, Doornfontein Campus, South Africa

**Keywords:** Metabolite profiling, Untargeted metabolites, GC-HRTOF-MS, Boiled walnut, Conophor nut

## Abstract

A variety of walnut known as *Tetracarpidium conophorum* is widely cultivated in several parts of Africa for its edible nuts. These nuts have been reported for their huge antioxidant, anti-obesity, and anti-depressant potentials, but remain underutilized due to their poor storage and preservation. This is why the nuts are mostly cooked and consumed as snacks whenever in season. This data article reports the untargeted metabolite profile of boiled and dried African walnut extracted using two different mixtures of solvents. The raw nuts obtained from a local market in Osun State, Nigeria, were processed by cooking for 20 min, deshelled, diced, dried at 60 ± 2 °C for 6 h, and stored until further analysis. The dried walnut samples were extracted with acetonitrile/methanol/water (40:40:20 v/v/v) and methanol/water (80:20 v/v) as solvents, before being analysed by gas chromatography high-resolution time of flight mass spectrometry (GC-HRTOF-MS) system. Data obtained from the analysis were further classified into different compounds, including alcohols, esters, hydrocarbons, phytosterols, vitamins, and many more. Their retention time, observed ion mass-to-charge ratio, molecular formula, and average peak areas were also reported. These data thus serve as a source of metabolites comparison for other walnuts, may be useful for the identification of functional compounds available in this neglected food crop, and encourage its utilization in developing functional foods.

**Specifications Table**

**Table:** 

Subject	Food Science: Food Chemistry
Specific subject area	Processing; Food composition and analysis; Metabolomics
Type of data	Table Figure Spectra
How the data were acquired	Raw walnuts were boiled under pressure for 20 min, sliced and dried at 60 ± 2 °C for 6 h. The dried cooked nuts were further grounded using laboratory mortar & pestle, and then extracted using two different combinations of organic solvents, acetonitrile/methanol/water (40:40:20 v/v/v), and methanol/water (80:20 v/v). The extracts were analyzed using the GC-HRTOF-MS system (LECO Pegasus, St Joseph, USA). This system featured 50,0 0 0 FWMH resolution (full peak with at one-half maximum), mass accuracies/errors of <1 ppm with acquisition rates up to 200 spectra/s, and was equipped with an Agilent 7890A gas chromatograph (Agilent Technologies, Inc., Wilmington, DE, USA). This GC-HRTOF-MS operates at high resolution and is equipped with a Gerstel MPS multipurpose autosampler (Gerstel Inc., Mülheim an der Ruhr, Germany) and a Rxi ®-5ms column (30 m × 0.25 mm ID × 0.25 µm) (Restek, Bellefonte, United States).
Data format	Raw data Analyzed data Filtered data Spectra of commonly identified compounds
Description of data collection	The already processed walnut (1 g) in its ground form was weighed and metabolites were extracted using the 10 mL mixture of different solvents (acetonitrile/methanol/water (40:40:20 v/v/v), and methanol/water (80:20 v/v) in each case. Thereafter, each sample was vacuum concentrated and reconstituted in chromatography-grade methanol (1 mL), then filtered with a 0.22 µm syringe into amber vials. Each sample (1 µL) was auto-injected into the GC-HRTOF-MS machine in triplicates and analyzed. The identities of the metabolite obtained were determined using NIST, Mainlib and Feihn metabolomics databases.
Data source location	Raw African walnuts were sourced from a local market in Modakeke, Osun State Nigeria (N 7°22’ 54.848” E 4°16’3.737”) on the 27th June, 2021 and processed within 24 h after collection. Thereafter, the extraction and analyses were carried out at the University of Johannesburg, Doornfontein, Johannesburg, Gauteng, South Africa (S 26°11’ 32.6” E 28°03’ 28.9”).
Data accessibility	Raw & processed dataset, and mass spectra of the metabolites have been deposited in the Mendeley repository. It is accessible using the details below: Repository name: Mendeley data. DOI: 10.17632/s9vrhj8tsk.1 Direct URL to data: https://data.mendeley.com/datasets/s9vrhj8tsk

## Value of the Data

• The data contributed to the identification of metabolites in African walnuts and provided information on the versatility of different solvent mixtures in metabolite extraction.
• The information provided herein will assist in understanding the usefulness of African walnuts and promote their cultivation to prevent crop extinction.
• The data could be useful for a comparative analysis of the metabolite composition in raw and processed, domestic or foreign walnuts, and the developed products.
• The data would be useful for food processors and researchers aiming to develop novel functional foods from African walnuts.
• The data would be useful in identifying constituents that may be responsible to sensory, functional, and nutritional effects and concentration in developed food products.
• The data would be useful resource for nutritionists, agronomists, food, and data scientists.
• The data indicates that untargeted GC-HRTOF-MS analysis could facilitate the identification of compounds that may be responsible for the nut's health-promoting effects.

## Objective

The African walnut (*Tetracarpidium conophorum*) plant has been extensively investigated for its high nutrients, anti-oxidants, anti-diabetic, anti-inflammatory, and other therapeutic benefits. The nut has also been explored in a few food products such as functional cookies [1], however, the nut has remained poorly utilized. This study identified metabolite present in ready-to-eat African walnuts that may enhance the potential exploitation of the nut as food ingredients in the development of functional food products that could solve many health issues.

## 1 Data Description

The dataset deposited in the repository contain two files (excel sheets and word document). The excel sheet 1 contains the raw data collected from the GC-HRTOF-MS analysis, it described the retention time (min), sample code, observed mass per charge number of ions, formula, area, name and synonym of the compounds extracted. Sheet 2 contains the data that was processed using the DataPrep solutions software and the class of the identified compounds, while sheet 3 represent the common compound that occurred at least two times in three injection in both samples analysed. The sample labelled W1 represent the walnut sample extracted with the mixture of acetonitrile/methanol/water (40:20:20 v/v/v), and W2 represent walnut sample extracted with the mixture of methanol/water (80:20 v/v). In addition, the word document deposited in the repository enclose the spectra of each compound identified in both samples. The spectrum of each compound typically shows a number of signals and the true peak at the highest mass per charge ion ratio. This will provide the scientist community with the structural information and the whole molecule identification.

**Fig. 1:**
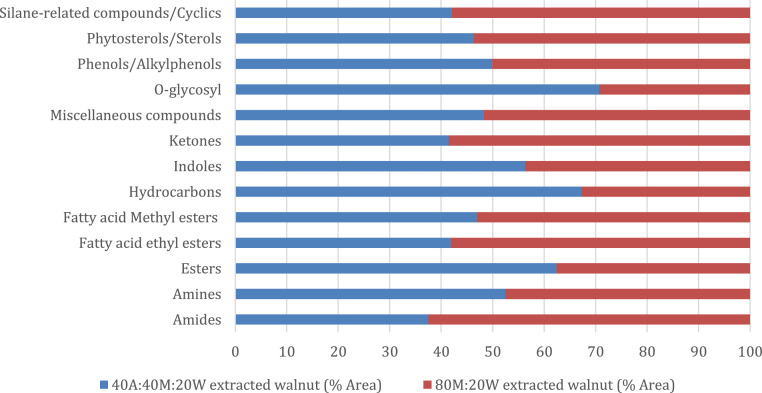
Percentage distribution of compounds common to both extracted walnut samples.

The metabolite data obtained from extracted walnut samples are presented below. Table 1 represents metabolites obtained from walnut extracted using the mixture of acetonitrile/methanol/water (40:20:20 v/v/v), and methanol/water (80:20 v/v) mixed solvent. The data in each table shows information regarding the name of each extractable compound identified, their retention time, observed ion mass-to-charge ratio, molecular formula and average peak area. These data were generated from GC-HRTOF-MS analysis and the spectra obtained were compared with NIST, Mainlib and Feihn metabolite databases. The raw and analyzed data along with the spectra of the identified compounds are available in a supplementary file deposited in the repository [2]. Figure 1 summarizes the percentage distribution of the compounds found from at least 2 out of 3 injections of each extracted sample from the extraction solvent.

**Table 1:** Metabolites identified in the walnut sample that was extracted using the two different solvents mixture.

Retention Time (Min)	Observed Ion *m/z*	Name	Molecular Formula	Average Area	
				W1	W2
**Acyclic alkanes**					

14.976	268.9873	Eicosane	C₂₀H₄₂	ND	135601

**Alcohols/Phenols**					

2.987	32.0259	Methyl Alcohol	CH₄O	2517321	ND
12.317	220.1821	Butylated Hydroxytoluene	C₁₅H₂₄O	ND	84268

**Aldehydes**					

7.576	120.0569	Benzaldehyde, 2-methyl-	C₈H₈O	698094	ND
15.962	234.1612	3,5-di-tert-Butyl-4-hydroxybenzaldehyde	C₁₅H₂₂O₂	ND	12759

**Amides**					

21.457, 21.742	142.1226, 156.1383	3-Cyclopentylpropionamide, N,N-dimethyl-	C₁₀H₁₉NO	180548	300932

**Amines**					

22.582, 22.580	144.1019, 144.1019	Bis(2-(Dimethylamino)ethyl) ether	C₈H₂₀N₂O	421813	382242

**Esters**					

21.490	225.4713	2-Propenoic acid, 3-(4-methoxyphenyl)-, 2-ethylhexyl ester	C₁₈H₂₆O₃	ND	19523
17.921, 17.918	292.2026, 292.2033	Benzenepropanoic acid, 3,5-bis(1,1-dimethylethyl)-4-hydroxy-, methyl ester	C₁₈H₂₈O₃	27667	32997
30.801	530.4694	Benzenepropanoic acid, 3,5-bis(1,1-dimethylethyl)-4-hydroxy-, octadecyl ester	C₃₅H₆₂O₃	ND	111822
22.581	219.0679	Carbonic acid, 2-dimethylaminoethyl 2-methoxyethyl ester	C₈H₁₇NO₄	ND	418022
21.606, 21.654	170.0830, 219.1150	Carbonic acid, 2-dimethylaminoethyl isobutyl ester	C₉H₁₉NO₃	316407	172290
25.181	297.2416	Decanedioic acid, bis(2-ethylhexyl) ester	C₂₆H₅₀O₄	ND	196860
23.307	279.1594	Dicyclohexyl phthalate	C₂₀H₂₆O₄	ND	67658
3.082	88.0519	Ethyl Acetate	C₄H₈O₂	ND	8575634
18.124	223.5891	Dibutyl phthalate	C₁₆H₂₂O₄	80025	ND
24.783, 24.780	279.1576, 279.1592	Mono(2-ethylhexyl) phthalate	C₁₆H₂₂O₄	81688	48734
25.443	503.1072	Phthalic acid, 8-chlorooctyl decyl ester	C₂₆H₄₁ClO₄	191547	ND
11.734	149.1072	Succinic acid, 3-methylbut-2-en-1-yl 3-methoxyphenyl ester	C₁₆H₂₀O₅	111337	ND
24.557	328.2897	Octadecanoic acid, 2,3-dihydroxypropyl ester	C₂₁H₄₂O₄	ND	646767
25.622	226.9907	Phthalic acid, 4-methylhept-3-yl pentyl ester	C₂₁H₃₂O₄	ND	53421

**Ethers**					

8.715, 12.656	131.1513, 131.1238	1,1,1,2,3,3,3-Heptafluoro-2-methoxypropane	C₄H₃F₇O	7819	7624
**Fatty acid ethyl esters (FAEEs)**					

22.989, 22.990	300.2605, 311.2588	Hexadecanoic acid, 2-hydroxy-1-(hydroxymethyl) ethyl ester	C₁₉H₃₈O₄	540587	751444

**Fatty Acid Methyl Esters (FAMEs)**					

19.473, 19.516	292.2395, 292.2398	9,12,15-Octadecatrienoic acid, (Z,Z,Z)-, Methyl 8,11,14-heptadecatrienoate	C₁₈H₃₀O₂	1476801	1179328
19.398, 19.398	294.2553, 294.2552	9,12-Octadecadienoic acid, methyl ester	C₁₉H₃₄O₂	1064918	1214274
21.261, 21.260	293.2822, 293.2820	9-Octadecenoic acid (Z)-, methyl ester	C₁₉H₃₆O₂	69858	151120
17.665, 17.663	270.2551, 270.2553	Hexadecanoic acid, methyl ester	C₁₇H₃₄O₂	2729756	2953203
19.674, 19.673	298.2860, 298.2868	Methyl stearate	C₁₉H₃₈O₂	1685892	2403367
23.094, 20.541	219.2037, 223.6918	Tridecanoic acid, methyl ester	C₁₄H₂₈O₂	197152	112146
23.035	199.1692	Undecanoic acid, methyl ester	C₁₂H₂₄O₂	118716	ND
19.451, 19.448	296.2707, 296.2708	trans-13-Octadecenoic acid, methyl ester	C₁₉H₃₆O₂	1003628	812770

**Hydrocarbons**					

13.420	131.1726	Butane, 1,1,1,2,3,3,4,4,4-nonafluoro-2-(trifluoromethyl)-	C₅F₁₂	6718	ND
15.032, 8.761	175.0624, 155.1432	Hexadecane	C₁₆H₃₄	84165	41015
11.656	139.0982	Pentadecane	C₁₅H₃₂	ND	79208

**Indoles**					

8.677, 8.680	117.0573, 117.0573	Indole	C₈H₇N	59297	46098

**Ketones**					

17.716, 17.710	276.1710, 267.0356	7,9-Di-tert-butyl-1-oxaspiro(4,5)deca-6,9-diene-2,8-dione	C₁₇H₂₄O₃	48189	94470
15.033, 15.032	188.1192, 219.1732	Methanone, (1-hydroxycyclohexyl)phenyl-	C₁₃H₁₆O₂	219837	287750
9.877	269.0488	7-Chloro-1,3,4,10-tetrahydro-10-hydroxy-1-[[2-[1-pyrrolidinyl]ethyl]imino]-3-[3-(trifluoromethyl)phenyl]-9(2H)-acridinone	C₂₆H₂₅ClF₃N₃O₂	ND	977127

**Miscellaneous compounds**					

19.981	292.2392	1,2-Benzenediol, O-(2-furoyl)-O'-(pentafluoropropionyl)-	C₁₄H₇F₅O₅	ND	160588
20.243	225.0656	1,8,11-Heptadecatriene, (Z,Z)-	C₁₇H₃₀	ND	273401
20.422	219.1343	1-Acetoxynonadecane	C₂₁H₄₂O₂	ND	75610
17.249	131.1923	1H-1,3-Benzimidazole-1-ethanol, a-(4-morpholinylmethyl)-	C₁₄H₁₉N₃O₂	ND	30635
24.268	131.0521	1H-Indole, 4-methyl-	C₉H₉N	ND	50238
13.318, 11.381	69.1009, 69.0252	2-Propynenitrile, 3-fluoro-	C₃FN	56110	10723
20.441	322.2496	3,4-Dimethoxybenzoic anhydride	C₁₈H₁₈O₇	ND	68270
11.937	503.1066	3-Isopropoxy-1,1,1,7,7,7-hexamethyl-3,5,5-tris(trimethylsiloxy)tetrasiloxane	C₁₈H₅₂O₇Si₇	ND	284860
13.434, 13.433	157.0884, 157.0882	3-Methyl-4-phenyl-1H-pyrrole	C₁₁H₁₁N	62006	50364
20.423	265.1814	Heneicosyl acetate	C₂₃H₄₆O₂	98265	ND
6.115	144.0419	Acetic acid, trifluoro-, ethyl ester	C₄H₅F₃O₂	ND	43042
23.999	269.0457	Anthranilic acid, 2TMS derivative	C₁₃H₂₃NO₂Si₂	ND	9488
6.517	357.0670	Cyclopentasiloxane, decamethyl-	C₁₀H₃₀O₅Si₅	ND	126744
19.172	219.1294	Dimethylmalonic acid, di(2-formylphenyl) ester	C₁₉H₁₆O₆	ND	3508
22.124	504.1076	Heptasiloxane, hexadecamethyl-	C₁₆H₄₈O₆Si₇	ND	176174
21.455	170.1539	Octanamide, N,N-dimethyl-	C₁₀H₂₁NO	ND	319657
14.843	210.0891	Methyl 3-(4-hydroxy-3-methoxyphenyl)propanoate	C₁₁H₁₄O₄	29002	ND
5.246	141.0699	Methyl 3-O-benzyl-alpha-d-glucofuranoside 5,6-carbonate	C₁₅H₁₈O₇	156833	ND
19.564, 19.562	503.1055, 505.1041	Octasiloxane, 1,1,3,3,5,5,7,7,9,9,11,11,13,13,15,15-hexadecamethyl-	C₁₆H₅₀O₇Si₈	118565	166258
13.576, 18.274	219.1379, 219.0369	Phosphine, tris(trifluoromethyl)-	C₃F₉P	4708	6508
6.721, 6.719	139.0991, 139.0991	Quinoline, decahydro-	C₉H₁₇N	200840	170716
13.535, 18.732	131.1894, 131.0855	Tris(trifluoromethyl) bromomethane	C₄BrF₉	6445	5364

**O-glycosyl**					

14.108, 14.093	217.1589, 137.0398	Ethyl a-d-glucopyranoside	C₈H₁₆O₆	352422	146265

**Phenols/Alkylphenols**					

12.252, 12.250	206.1660, 206.1658	2,4-Di-tert-butylphenol	C₁₄H₂₂O	537673	454311
22.354, 22.352	340.2395	Phenol, 2,2′-methylenebis[6-(1,1-dimethylethyl)-4-methyl-	C₂₃H₃₂O₂	1244321	1333882

**Phenylpropanes**					

27.948	278.0431	4-tert-Octylphenol, TMS derivative	C₁₇H₃₀OSi	1089432	ND

**Phytosterols/Sterols**					

29.015, 29.010	412.3703, 412.3677	Stigmasta-5,24(28)-dien-3-ol, (3ß,24Z)-	C₂₉H₄₈O	274827	248085
28.506, 28.504	412.3700, 412.3699	Stigmasterol	C₂₉H₄₈O	452029	599436

**Pyrazine/Pyridines**					

4.866	123.0679	4(H)-Pyridine, N-acetyl-	C₇H₉NO	593145	ND
19.154	116.0705	1,4-Di(methyl-d3)benzene-d4	C₈D₁₀	ND	72152

**Sesquiterpenoids**					

12.023	204.1864	(1R,5R)-2-Methyl-5-((R)-6-methylhept-5-en-2-yl)bicyclo[3.1.0]hex-2-ene	C₁₅H₂₄	ND	52683
10.838	199.9870	Bicyclo[7.2.0]undec-4-ene, 4,11,11-trimethyl-8-methylene-,[1R-(1R*,4Z,9S*)]-	C₁₅H₂₄	ND	28580
**Silane-related compounds/Cyclics**					

21.020	432.0861	1,1,1,5,7,7,7-Heptamethyl-3,3-bis(trimethylsiloxy)tetrasiloxane	C₁₃H₄₀O₅Si₆	ND	184425
18.940, 14.547	504.1078, 415.0368	Cyclooctasiloxane, hexadecamethyl-	C₁₆H₄₈O₈Si₈	132164	214475
28.287	221.0456	Cyclotrisiloxane, hexamethyl-	C₆H₁₈O₃Si₃	3083849	ND
8.916, 8.915	432.0872, 432.0848	Cyclohexasiloxane, dodecamethyl-	C₁₂H₃₆O₆Si₆	315246	401864

**Trialkylheterosilanes**					

11.938	503.1089	3-Isopropoxy-1,1,1,7,7,7-hexamethyl-3,5,5-tris(trimethylsiloxy)tetrasiloxane	C₁₈H₅₂O₇Si₇	198812	ND

**Vitamins**					

26.254, 26.253	402.3488, 402.3486	d-Tocopherol	C₂₇H₄₆O₂	1045910	686038

ND: Not detected; *m/z*: mass-to-charge ratio.

W1: Walnut sample extracted with acetonitrile/methanol/water (40:20:20 v/v/v); W2: Walnut sample extracted with methanol/water (80:20 v/v).

## 2 Experimental Design, Materials and Methods

### 2.1 Walnut collection and processing

Matured raw walnuts (*Tetracarpidium conophorum*) were sourced from a local market in Nigeria (N 7°22’ 54.848” E 4°16’3.737”) on the 27th June, 2021. They were physically cleaned and washed under running water to remove extraneous materials. The nuts were cooked (Pressure Pot, Master Chef, 12L) for about 20 minutes after pressure has been built within the system. The nuts were allowed to cool, de-shelled, and shredded into smaller sizes using a hand grater. The grated walnuts were dried in a food dehydrator (Bosch BS-6605, Germany) set at a temperature of 60 ± 2 °C for 6 h. The dried walnut was allowed to stand at room temperature before milling (Perten 3600, Sweden) to a coarse powder.

### 2.2 Metabolites extraction from the samples and analysis using GC-HRTOF-MS

The cooked walnut powder sample was extracted using two different mixtures of extraction solvents, acetonitrile/methanol/water (40:40:20 v/v/v) and methanol/water (80:20 v/v), following the method previously described by Oyedeji et al. [3]. One (1) gram of each of the walnut samples was weighed separately into 50 mL centrifuge tubes, 10 mL of each extraction solvent was added, and vortexed (Vortex-Gernie K-550-GE, Bohemia USA) vigorously to ensure even mixing. The tube containing the mixture was sonicated (Ultrasonic AU-200 Argo Lab, Italia Italy) for an hour, and then centrifuged (Eppendorf 5702R, Merck, Modderfontein South Africa) for 5 min at 4°C and 3500 rpm. The supernatants from each centrifuge tube were decanted into new tubes, and allowed to dry in a vacuum concentrator (Eppendorf Plus, Merck, Modderfontein South Africa). These recovered dried extracts were reconstituted with 1 mL chromatography-grade methanol (99.9% pure), and vortexed to ensure there is even dissolution of the extracts in each tube. The extracts was filtered into dark amber vials using PTFE-L 0.22 µm. Using the Pegasus GC-HRTOF-MS system (LECO Corporation, St. Joseph, MI, USA) with a resolution of 50,0 0 0 FWMH (full peak with at one-half maximum), mass accuracies/errors of < 1 ppm and acquisition rates of up to 200 spectra/s, the samples were analysed. This analytical system was equipped with a multipurpose sampler (Gerstel Inc., Mülheim an der Ruhr Germany) and Rxi ®-5 ms column (30 m × 0.25 mm ID × 0.25 µm) (Restek, Bellefonte, USA). An aliquot of each sample was injected without spit and pumped with helium as the carrier gas at a constant flow rate of 1 mL/min. Inlet and transfer line temperatures were set at 250 and 225${\;}^{\circ }C$ respectively and the ion source temperature was at 250${\;}^{\circ }C$. The oven temperature cycle used was: 70${\;}^{\circ }C$, 0.5 min for initial temperature; then an increase form 10${\;}^{\circ }C$/min to 150${\;}^{\circ }C$ for 2 min; then ramped up to 330 °C at 10 °C/min and held for 3 min to allow the column to ‘bake-out’. The solvent blanks were also tested in parallel to monitor for potential impurities and contamination. When processing the raw data with DataPrep solutions, parameters such as a signal-to-noise ratio of 50, a similarity match of over 70 % and at least twofold occurrence of metabolites from the triplicate data were strictly considered. The properties of the metabolites were identified by matching the spectra to NIST, Mainlib, and Feihn reference library databases. Data obtained from samples extracted with acetonitrile/methanol/water (40:40:20 v/v/v) and methanol/water (80:20 v/v) are presented in Table 1, and commonly detected compounds are summarised in Fig. 1. The raw and processed data, are presented in the supplementary file along with the raw spectra of some identified compounds.

## Ethics Statements

This work does not involve chemicals, procedures or equipment that have any unusual hazards inherent in their use, and it does not involve human subjects, animal experiments, or any data collected from social media platforms.

## CRediT Author Statement

**Beatrice Mofoluwaso Oladimeji:** Conceptualization, Sample preparation, Formal data analysis, Methodology, Visualization, Validation, Writing- original draft; **Oluwafemi Ayodeji Adebo:** Conceptualization, Funding acquisition, Data curation, Methodology, Resources, Software, Visualization, Supervision, Writing –review & editing.

## Funding

This work was supported financially by the 10.13039/501100006565University of Johannesburg (UJ) Global Excellence and Stature (GES 4.0) grant offered to Beatrice M. Oladimeji, and the UJ Research Committee (URC 2022) research grant awarded to Oluwafemi Ayodeji Adebo.

## Declaration of Competing Interest

The authors declare that they have no known competing financial interests or personal relationships that could have appeared to influence the work reported in this paper.

## Data Availability

Supplementary data for manuscript on metabolites extracted from African walnut (Tetracarpidium conophorum) using two different solvents (Original data) (Mendeley Data)
